# The Inhibition Effect and Mechanism of Staurosporine Isolated from *Streptomyces* sp. SNC087 Strain on Nasal Polyp

**DOI:** 10.3390/md22010039

**Published:** 2024-01-11

**Authors:** Grace Choi, Eun-Young Lee, Dawoon Chung, Kichul Cho, Woon-Jong Yu, Sang-Jip Nam, Seong-Kook Park, Il-Whan Choi

**Affiliations:** 1 Department of Microbial Resources, National Marine Biodiversity Institute of Korea, Seocheon 33662, Republic of Korea; dwchung@mabik.re.kr (D.C.); kichul.cho@mabik.re.kr (K.C.); woonjong_yu@mabik.re.kr (W.-J.Y.); 2 Department of Chemistry and Nanoscience, Ewha Womans University, Seoul 03760, Republic of Korea; younglee0124@naver.com (E.-Y.L.); sjnam@ewha.ac.kr (S.-J.N.); 3 Department of Otorhinolaryngology-Head & Neck Surgery, Busan Paik Hospital, Inje University College of Medicine, Busan 47392, Republic of Korea; sinus4@paik.ac.kr; 4 Department of Microbiology and Immunology, Inje University College of Medicine, Busan 47392, Republic of Korea

**Keywords:** staurosporine, nasal polys, ECM proteins, myofibroblast differentiation, *Streptomyces* sp. SNC087

## Abstract

This study aims to explore the potential inhibition effects of staurosporine isolated from a *Streptomyces* sp. SNC087 strain obtained from seawater on nasal polyps. Staurosporine possesses antimicrobial and antihypertensive activities. This research focuses on investigating the effects of staurosporine on suppressing the growth and development of nasal polyps and elucidating the underlying mechanisms involved. The experimental design includes in vitro and ex vivo evaluations to assess the inhibition activity and therapeutic potential of staurosporine against nasal polyps. Nasal polyp-derived fibroblasts (NPDFs) were stimulated with TGF-β1 in the presence of staurosporine. The levels of α-smooth muscle actin (α-SMA), collagen type-I (Col-1), fibronectin, and phosphorylated (p)-Smad 2 were investigated using Western blotting. VEGF expression levels were analyzed in nasal polyp organ cultures treated with staurosporine. TGF-β1 stimulated the production of Col-1, fibronectin, and α-SMA and was attenuated by staurosporine pretreatment. Furthermore, these inhibitory effects were mediated by modulation of the signaling pathway of Smad 2 in TGF-β1-induced NPDFs. Staurosporine also inhibits the production of VEGF in ex vivo NP tissues. The findings from this study will contribute to a better understanding of staurosporine’s role in nasal polyp management and provide insights into its mechanisms of action.

## 1. Introduction

Nasal polyps (NPs), also known as sinus polyps, are abnormal protrusions of the nasal mucosa that result from recurring local inflammation and gradually develop into a benign swelling of the mucous membranes in the nasal cavity and paranasal sinuses [1]. The nasal polyp (NP) mucosa is mostly composed of respiratory epithelium, and the submucosal layer shows significant swelling, with the infiltration of inflammatory cells, particularly eosinophils [2]. The exact cause of NPs is not fully understood, but there have been reports suggesting a potential association with allergic reactions, inflammation, epithelial immune barrier dysfunction, and genetic factors [1,3,4]. While the precise underlying mechanisms remain unclear, these factors are believed to play a role in the development of NPs. It is important to note that these factors are not the sole causes of NPs, and other environmental and individual factors may also contribute to their development. Further research is needed to fully understand the complex mechanisms underlying the formation of NPs.

The treatment of NPs aims to alleviate symptoms caused by the polyps, restore normal nasal function, promote the drainage and ventilation of the sinuses, and prevent recurrence. Treatment options for NPs can be broadly categorized into medication therapy and surgical intervention [5]. Medication is now known to be effective in treating NPs, where topical corticosteroid sprays are commonly prescribed to reduce inflammation, shrink polyps, and relieve symptoms such as nasal congestion and a runny nose. In some cases, corticosteroids can be injected directly into the polyps to reduce their size; this approach may beneficial for larger polyps [6]. As another treatment, if the polyps are large or do not respond to medication therapy, surgical removal may be necessary. The goal of surgery is to completely remove the polyps, restore nasal airflow, and improve sinus drainage. Surgical procedures can be performed using traditional open techniques or endoscopic approaches, where a thin tube with a camera is inserted through the nostrils to visualize and remove the NPs [7]. However, since remodeling of NPs is common after removal surgery, many patients require additional procedures after NPs recurrence. It is important to note that NPs are often associated with chronic sinusitis. Therefore, managing and treating a sinus inflammation is also an integral part of the overall treatment. Additional medication therapies or treatment modalities may be employed to address sinus inflammations, and a combination of medication therapy and surgery is often used in practice [1,7].

Many types of immune cells are involved in NPs pathogenesis, including T cells, goblet cells, epithelial cells, eosinophils, mast cells, lymphocytes, and fibroblasts [8,9]. Among these cells present in NPs, fibroblasts are the cell type of the NP architecture that contributes to the formation of NPs [10]. Fibroblasts are connective tissue cells in all body tissues [11]. They produce and react to various inflammatory cytokines [12]. Fibroblasts play a biological role in wound healing, tissue remodeling, angiogenesis, and inflammation [13,14]. They are the central mediators of the accumulation of extracellular matrix (ECM), and cell differentiation and proliferation are caused by long-term tissue damage [15]. These tissue injuries often stimulate the differentiation of fibroblasts to myofibroblasts, which are involved in the inflammatory reaction to injury [16]. When activated by profibrotic stimulation, fibroblasts are differentiated into myofibroblasts, resulting in the production of excessive ECM proteins, such as collagen and fibronectin [17]. The differentiation of fibroblasts into myofibroblasts corresponds to the physiological process that promotes the formation of NPs [18]. The fibroblasts present in the NP stroma are NP-derived fibroblasts (NPDFs). The expression of smooth muscle actin is a reliable indicator of myofibroblast differentiation. It has been reported that damage to the mucosal epithelium results in the expression of transforming growth factor-β1 (TGF-β1) [19]. Increased accumulation of ECM components, mobility and invasion, migration and accumulation, angiogenesis and immune response characterize TGF-β1stimulation [20,21]. Furthermore, TGF-β1 is an important profibrotic cytokine associated with fibroblast activation and differentiation [22]. NPs have shown that they have high levels of TGF-β1 [23].

Staurosporine (STA) was initially isolated from *Streptomyces staurosporeus* in 1977 [24]. It is known to possess antimicrobial and antihypertensive activities [25]. The primary biological activity of STA is the inhibition of protein kinase C by preventing ATP from binding to the kinase [25,26]. This is achieved through its strong affinity for the ATP-binding site of the kinase. Additionally, STA induces cell apoptosis by activating caspase-3, leading to the arrest of cell division in the G1 or G2 phase [26,27]. Due to STA’s ability to induce apoptosis in several cell lines, including cervical, colon, oral, and breast cancers, it has been studied for its potential antitumor activity in vitro [28,29,30,31]. Past clinical and preclinical studies using STA analogs, UCN-01, lestaurtinib, and midostaurin, have been tested in several clinical trials for various cancer subtypes, including leukemia, lymphoma, melanoma, and solid tumors. Among these, midostaurin is still undergoing active clinical trials [32]. In clinical studies, liposome nanoparticles encapsulating STA demonstrated anticancer effects without side effects in mouse experiments [33]. While various beneficial effects of STA have been reported through research, there is currently no specific evidence regarding its potential improvement in the treatment of NPs. In this study, we aim to introduce the inhibition effect of STA obtained from marine bacteria isolated from seawater, specifically focusing on its effects on NPs and its underlying mechanisms.

## 2. Results and Discussion

### 2.1. Isolation and Identification of Strain SNC087

The pure isolate of strain SNC087 exhibited morphological characteristics similar to those of typical strains belonging to the genus streptomyces. For the species identification of the strain, a 1 mL culture of the SNC087 strain, cultured in SYP liquid medium at 27 °C for 5 days, was used to extract genomic DNA using the “Tissue GenomeDNA Isolation Kit” (Cosmogenetech Co., Ltd., Seoul, Republic of Korea) following the manufacturer’s protocol. For 16S rRNA gene amplification for species analysis, PCR was performed using 27F and 1492R primers. The PCR products were purified using the “PCR purification kit” (Cosmogenetech co, Ltd., Seoul, Republic of Korea), and the nucleotide sequence was analyzed using capillary electrophoresis (Applied Biosystems 3730XL) for base sequencing. The obtained 16S rRNA gene sequence from SNC087 strain was compared to the information of previously reported strains using BLAST search in the GenBank/EMBL/DDBJ databases. As a result, the 16S rRNA gene sequence of SNC087 strain showed 99.9% similarity with the *Streptomyces sanyensis* 219820 strain (accession no. FJ261968), indicating that the SNC087 (accession no. MK850320) strain belongs to the genus Streptomyces.

### 2.2. Structural Elucidation of STA

**Figure 1:**
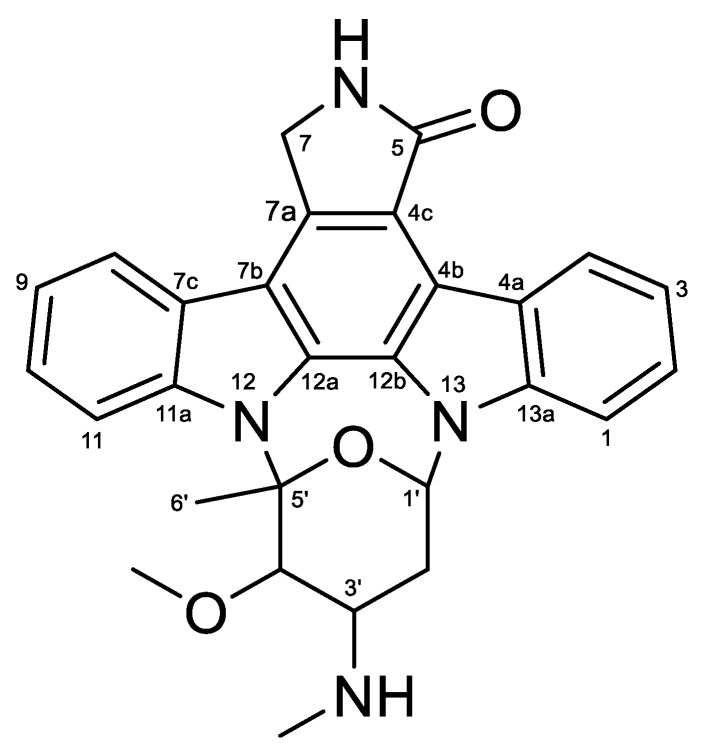
Chemical structure of **1** (Staurosporine, STA).

Compound **1** was isolated as light yellow powder, and LR-ESI-MS spectroscopy revealed an ionic peak at *m*/*z* 467.3 [M+H]^+^. The HPLC-UV-guided isolation of this extract led to the isolation of **1**. The ^1^H NMR spectrum of **1** showed three aromatic protons (*δ*_H_ 9.39 (d, *J* = 8.2 Hz, H-4), *δ*_H_ 7.89 (overlapped, H-8 and H-11), *δ*_H_ 7.48 (d, *J* = 7.1 Hz, H-1), *δ*_H_ 7.35 (overlapped, H-2 and H-10), and *δ*_H_ 7.30 (overlapped, H-3 and H-9)), two methine protons (*δ*_H_ 6.69 (brs, H-1′) and *δ*_H_ 3.35 (m, H-3′)), two methylene protons (*δ*_H_ 4.99 (s, H-7), and *δ*_H_ 1.63 (brs, H-2′)), one methyl proton (*δ*_H_ 1.26 (s, H-6′)), a methoxy group proton (*δ*_H_ 3.92 (s, 4′-OCH_3_)), and an N-methyl group proton (*δ*_H_ 2.36 (s, 3′-NCH_3_)). ^13^C NMR of **1** also indicated twenty eight carbon signals, including one carbonyl carbon (C-5 (*δ*_C_ 173.7)), eight aromatic carbons (C-4 (*δ*_C_ 126.7), C-2 (*δ*_C_ 125.2), C-10 (*δ*_C_ 124.8), C-8 (*δ*_C_ 120.8), C-9 (*δ*_C_ 120.1), C-3 (*δ*_C_ 119.9), C-11 (*δ*_C_ 115.4), and C-1 (*δ*_C_ 107.1)), ten fully substituted carbons (C-11a (*δ*_C_ 1739.9), C-13a (*δ*_C_ 136.8), C-7a (*δ*_C_ 132.3), C-12a (*δ*_C_ 130.9), C-12b (*δ*_C_ 127.3), C-7c (*δ*_C_ 124.3), C-4a (*δ*_C_ 123.7), C-4c (*δ*_C_ 118.6), C-7b (*δ*_C_ 115.4), and C-4b (*δ*_C_ 114.2)), four methine carbons (C-5′ (*δ*_C_ 91.3), C-4′ (*δ*_C_ 84.2), C-1′ (*δ*_C_ 80.3), and C-3′ (*δ*_C_ 57.4)), two methylene carbons (C-7 (*δ*_C_ 50.5) and C-2′ (*δ*_C_ 14.3)), once methyl carbon (C-6′ (*δ*_C_ 21.2)), one methoxy carbon (4′-OCH_3_ (*δ*_C_ 60.5)) and one N-methyl carbon (3′-OCH_3_ (*δ*_C_ 21.2)) (Figures S1 and S2). Based on the comparison of NMR data of **1** with those reported, compound **1** was identified as STA (Figure 1) [34].

### 2.3. Toxicity of STA in Nasal Polyp-Derived Fibroblasts (NPDFs)

**Figure 2:**
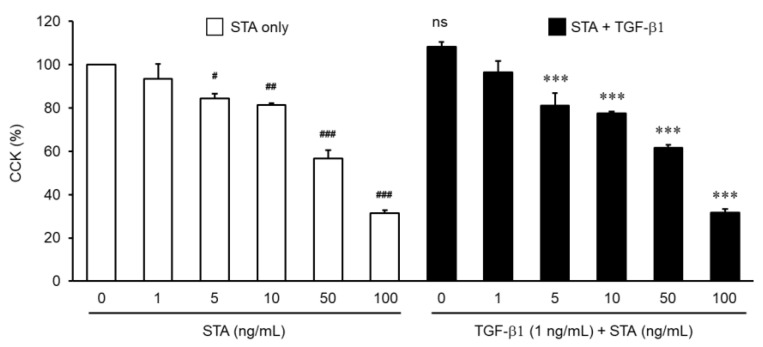
Effects of staurosporine (STA) toxicity on NPDF. The cells were treated for 24 h at different concentrations (1, 5, 10, 50, and 100 ng/mL) of STA in the absence or presence of 1 ng/mL TGF-*β*1. The cell viability was assessed using the Cell Counting Kit-8 (CCK-8) test. The results are expressed as the percentage of survived cells relative to the percentage of untreated cells. Each value indicates means ± standard error of the mean (SEM) and represents the results of three independent experiments. ns vs. control group (no treatment group); **^#^** *p* < 0.05, ^##^ *p* < 0.01, and ^###^ *p* < 0.001 vs. control group (no treatment group); *** *p* < 0.001 vs. TGF-*β*1-stimulated group.

First, in order to understand the antifibrosis effects of STA, we studied the effects of STA toxicity on the NPDFs. NPDFs were treated with STA at a concentration of 1–100 ng/mL. Toxicity tests have shown that STA does not cause NPDF cytotoxicity below 10 ng/mL (Figure 2). Because more than 40% toxicity was observed at concentrations above 50 ng/mL, concentrations below 10 ng/mL were used. Based on these results, follow-up experiments with STA were conducted in the range 1–10 ng/mL.

### 2.4. Effect of STA on the Expression of α-SMA, Col-1, and Fibronectin in TGF-β1-Activated NPDFs

**Figure 3:**
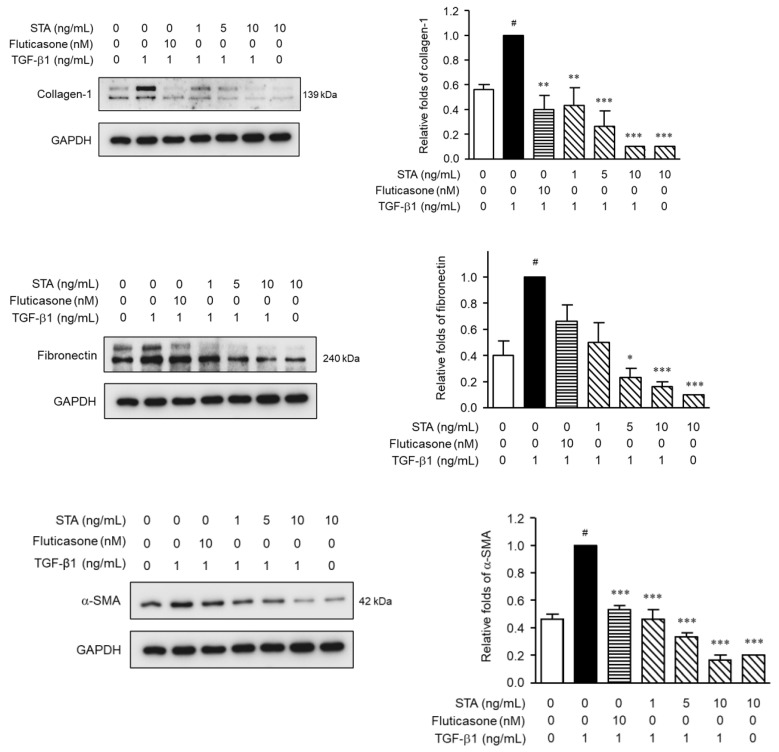
Effect of STA on *α*-SMA, Col-1, and fibronectin proteins the expression levels in fibroblasts produced by TGF-*β*1 stimulated by nasal polyp-derived fibroblasts. The cells were planted at 2 *×* 10^5^ cells/mL and incubated for 1 h before TGF-*β*1 stimulation at different STA concentrations (1, 5, and 10 ng/mL). After stimulation with TGF-*β*1 for 3 h, the expression of proteins SMA, Col-1, and fibronectin was determined via Western blot analysis. GAPDH was used as the internal control. The data are means ± standard error of the mean (SEM) and are representative of the results obtained in three independent experiments. ^#^ *p* < 0.05 vs. control group (no treatment group); * *p* < 0.05, ** *p* < 0.01, and *** *p* < 0.001 vs. TGF-*β*1-stimulated group.

Next, we investigated whether STA suppressed the TGF-β1-induced expression of α-SMA, collagen type-I (Col-1), and fibronectin in TGF-β1-stimulated NPDFs. Fibroblasts and myofibroblasts are well-known to play an important role in the remodeling process associated with chronic rhinovirus with NP (CRSwNP) [35]. Myofibroblasts are well established in NPs, but are almost absent from normal turbinated tissue. Thus, myofibroblasts play an important role in the pathogenesis of NP. It is well known that the accumulation of ECM proteins (Col-1 and fibronectin) and myofibroblast (α-SMA) differentiation is an important factor in the pathogenic development of NP formation [36,37]. α-SMA expression is the defining feature of mature myofibroblasts and has been shown to increase the contraction activity level of fibroblasts [38]. Fibronectin is a multifunctional glycoprotein involved in tissue remodeling and is known as a chemoattractant of the fibroblast; it can be released in larger amounts by the fibroblast in response to various cytokines [39]. Collagen is an important structural protein in the extracellular space of various animal connective tissues. Collagen plays a role in growth, differentiation, and wound healing. Significantly, the expression level of Col-1 deposited in NP increased compared to that of the nasal turbinates in normal subjects [40]. Damage to the mucous membrane epithelium induces TGF-β1 expression. TGF-β1 expression in NP tissue is at a high level, accompanied by structural changes characterized by NP formation [38]. Our previous studies have shown that TGF-β1 increases the α-SMA, fibronectin, and Col-1 protein levels in NPDFs [38]. Therefore, restricting the expression of fibrosis-promoting mediators is one of the important means of blocking NP remodeling. As expected, the expression levels of α-SMA, fibronectin, and Col-1 in NPDFs were significantly increased by the stimulation of TGF-β1 in our experimental system. Therefore, we investigated the inhibitory effects of STA on the differentiation of myofibroblasts and the expression of ECM proteins in non-cytotoxic concentrations (1, 5, and 10 ng/mL) of NPDFs stimulated with TGF-β1. Fluticasone is used to treat severe symptoms of NPs [41]. Therefore, fluticasone was chosen as a positive control in our experimental system. STA was administered to the cells 1 h before 24 h of TGF-β1 stimulation. As shown in Figure 3, the expression of α-SMA, Col-1, and fibronectin stimulated by TGF-β1 was suppressed due to the STA treatment. These results indicate that STA inhibits the differentiation of myofibroblasts and the production of ECM proteins in TGF-β1-stimulated NPDFs. Consequently, STA suggests that it may be a potential treatment for inhibiting NP remodeling after surgery.

### 2.5. Regulatory Mechanisms of the Fibrosis Inhibition by STA in TGF-β1-Activated NPDFs

**Figure 4:**
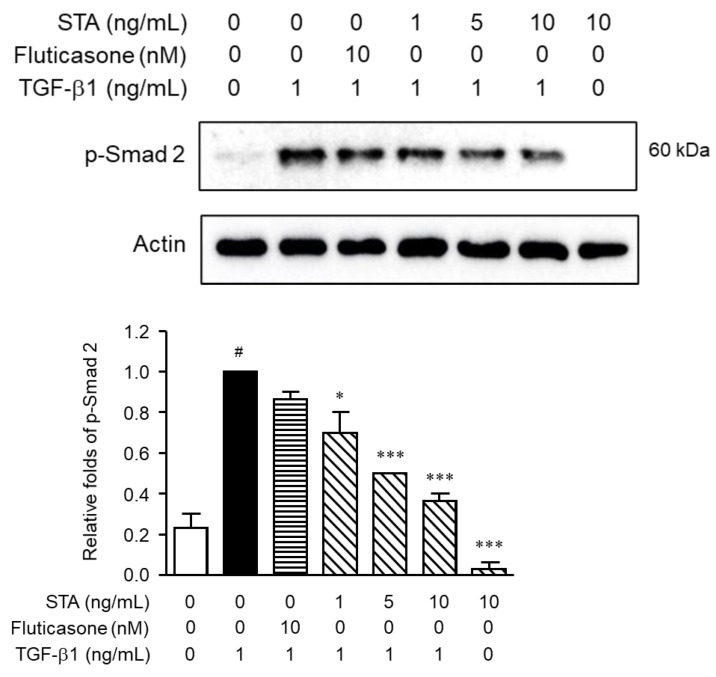
Effect of STA on phosphorylation of Smad 2 in NPDFs caused by TGF-β1. Cells were treated with the indicated STA concentration (1, 5, and 10 ng/mL) for 1 h, followed by stimulation with TGF-β1 (1 ng/mL) for 1 h, and a nuclear protein was extracted. The nuclear protein was subjected to a Western blot with antibodies specific for the phosphorylated forms of Smad 2. The data are means ± standard error of the mean (SEM) and are representative of the results obtained in three independent experiments. ^#^ *p* < 0.05 vs. control group (no treatment group); * *p* < 0.05 and *** *p* < 0.001 vs. TGF-*β*1-stimulated group.

To understand the signal pathways underlying the inhibitory effects of STA on NPDFs’ fibrogenic responses, we examined key signal pathways mediated by TGF-β1. The Smad pathway, a canonical signal pathway, is a critical mechanism for signals from TGF-β1 receptors [37]; therefore, we evaluated the effects of STA on Smad 2 phosphorylation (p-Smad 2). When TGF-β1 binds to receptors, such as type I and type II TGF receptors, the downstream signal Smad is activated. TGF-β1 binds to the receptor and induces the phosphorylation of Smad 2. Active Smad 2 forms oligomeric complexes with Smad 4, which move to the nucleus [42]. These complexes activate the transcription of profibrotic genes and induce fibrotic reactions. In this study, p-Smad 2 was significantly enhanced in NPDFs stimulated with TGF-β1 (Figure 4). However, the p-Smad 2 expression levels were suppressed when the NPDFs were treated for 1 h with STA (1, 5, and 10 ng/mL) before stimulation with TGF-β1 for 1 h. These results show that STA inhibits the production of ECM proteins and the differentiation of myofibroblasts through the Smad 2 pathway in NPDFs stimulated with TGF-β1.

### 2.6. Measurement of Expression Inhibition Efficacy of VEGF (Vascular Endothelial Growth Factor) Using NPs (Ex Vivo)

Several studies reported a high level of Vascular Endothelial Growth Factor (VEGF) in the epithelium and endothelium of NPs [43,44]. VEGF is a 45 kDa homodimeric glycoprotein that binds heparin and plays an important role in angiogenesis and vessel remodeling. VEGF induces endothelial cell proliferation, increases vascular permeability, and participates in wound healing, tumor growth, and chronic inflammation [45]. VEGF may lead to increased vessel permeability, and finally, to the known characteristic tissue edema of NPs. Histologically observed hallmarks of remodeling are macrophage and lymphocyte migration, fibroblast proliferation, angiogenesis, subepithelial fibrosis, and tissue degeneration [46]. Angiogenesis and microvascular remodeling are components of the tissue remodeling of NPs [47]. Thus, the negative regulators of angiogenesis are required to block NPs remodeling. 

**Figure 5:**
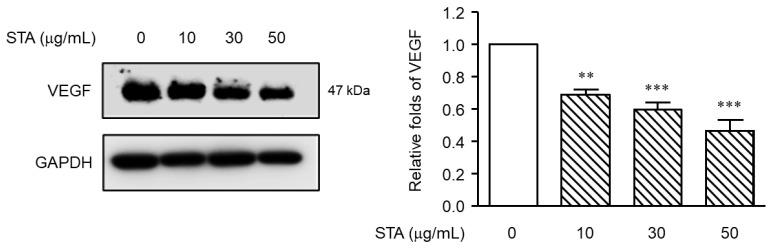
Effect of STA on VEGF expression levels in nasal polyp tissue. The NP tissues were treated with STA (10, 30, and 50 μg/mL) using an air–liquid interface organ culture method. After 24 h of incubation with STA, the level of expression of VEGF protein was assessed by Western blotting. Each GAPDH is used for internal control. The data are means ± standard error of the mean (SEM) and are representative of the results obtained in three independent experiments. ** *p* < 0.01 and *** *p* < 0.001 vs. control group (no treatment group).

We used an organ culture model of the air–liquid interface. The tissue culture requires efficient gas diffusion and the exchange of metabolites and nutrient supplies [48]. Cultivation at the air–liquid interface helps to solve these problems by promoting the exchange of gases, while maintaining nutrient access. Furthermore, the structural integrity and biochemical activity of both the submucosal and epithelial components can be maintained. These air–liquid interface culture models show that various cells, such as surface epithelial cells, inflammatory cells, and endothelial cells, have the ability to express vascular endothelial growth factors. To address this issue, a primary NP culture was performed to investigate the anti-angiogenic efficacy of STA to block angiogenesis required for NP remodeling. Using an organ culture model, we investigated whether STA reduced the VEGF expression in NP tissues. As shown in Figure 5, the levels of VEGF were effectively reduced depending on the dose of the STA. This result suggests modulating VEGF expression using STA can be a therapeutic strategy for managing NP recurrence after endoscopic sinus surgery.

## 3. Materials and Methods

### 3.1. Chemical and Reagents

NMR spectra were acquired by containing Me_4_Si as internal standard using Varian Inova spectrometers, 400 MHz and 100 MHz spectrometers (Varian Medical Systems, Inc., Charlottesville, VA, USA), using solvent chloroform-*d* (Cambridge Isotope Laboratories (CIL), Inc., Tewksbury, MA, USA). Low-resolution LC-MS measurements were taken using the Agilent Technology 1260 quadrupole (Agilent Technologies, Santa Clara, CA, USA) and Waters Alliance Micromass ZQ LC-MS system (Waters Corp, Milford, MA, USA) using a reversed-phase column (Phenomenex Luna C18 (2) 100 Å, 50 × 4.6 mm, 5 µm) (Phenomenex, Torrance, CA, USA) at a flow rate 1.0 mL/min at the National Research Facilities and Equipment Center (NanoBioEnergy Materials Center, Argonne, IL, USA) at Ewha Womans University.

TGF-β1 was purchased from R&D Systems, Inc. (Minneapolis, MN, USA). We used Cell Counting Kit-8 (CCK-8) manufactured by DOJINDO Laboratories (Rockville, MD, USA). We purchased antibodies against α-SMA and Col-1 from Abcam (USA; catalog number ab5694 and ab88147, respectively). Antibodies against actin and fibronectin were purchased from BD Biosciences (San Jose, CA, USA; cat. no. 612,656 and 610077, respectively). Antibodies against GAPDH and goat anti-mouse IgG (HRP) conjugate were purchased from Young In Frontier (Seoul, Korea, cat. no. LF-PA0018 and LF-SA8001, respectively). 

### 3.2. Strains and Culture Conditions

We isolated pure strains from seawater collected near Il-gwang Beach in Gijang-gun, Busan. This seawater was inoculated onto Mar4 solid media (2 g kelp meal, 2 g D-mannitol, 1 g fish meal, 20 g/L KBr, 8 g/L Fe_2_(SO_4_)_3_·4H_2_O, 30 mL DMSO, 970 mL filtered seawater, and 21 g agar) and cultured at 27 °C. Subsequently, a single strain was isolated and selected by re-inoculating it onto SYP solid media (10 g starch, 4 g yeast, 2 g peptone, 1 L filtered seawater, and 21 g agar) to obtain a pure bacterial strain. 

Strain SNC087 was cultured in 36 × 2.5 L Ultra Yield flask each containing 1 L of medium (10 g/L soluble starch, 4 g/L yeast, 2 g/L peptone, and 34.75 g/L artificial sea salt dissolved in distilled H_2_O) at 27 °C and constantly shaken at 120 rpm. After seven days, the broth was extracted with ethyl acetate (EtOAc) (36 L overall), and the EtOAc-soluble fraction was dried in vacuo to yield 1.23 g of organic extract.

### 3.3. Isolation and Purification of STA from the SNC087 Strain

The crude extract of SNC087 (1.23 g) was fractionated using a C-18 resin MPLC column (Biotage SNAP Cartridge, KP-SIL) and eluted with a step gradient from 0 to 100% MeOH in H_2_O to obtain 10 fractions (SCN087-1−SNC087-10). Subfraction eight was isolated using reversed-phase HPLC (Reprosil 100 C-18 10 μm 250 × 20 m, L 250, 7.0 mL/min, and UV = 254 nm) using an isocratic condition 65% MeOH in H_2_O to obtain STA (**1**, 41.2 mg, t*_R_* = 15.5 min).

*Staurosporine* (**1**): light yellow powder; ^1^H NMR (400 MHz, CDCl_3_): *δ*_H_ 9.39 (d, *J* = 88.2 Hz, H-4), 7.89 (overlapped, H-8 and H-11), 7.48 (d, *J* = 7.1 Hz, H-1) 7.35 (overlapped, H-2 and H-10), 7.30 (overlapped, H-3 and H-9), 6.69 (brs, H-1′), 4.99 (s, H-7), 3.92 (s, H-4′), 3.35 (m, H-3′), 2.36 (s, 3′-NCH_3_), 1.63 (brs, H-2′), 1.26 (s, H-6′); ^13^C NMR (100 MHz, CDCl_3_): *δ*_C_ 173.7 (C-5), 139.9 (C-11a), 136.8 (C-13a), 132.3 (C-7a), 130.9 (C-12a), 127.3 (C-12b), 126.7 (C-4), 125.2 (C-2), 124.8 (C-10), 124.3 (C-7c), 123.7 (C-4a), 120.8 (C-8), 120.1 (C-9), 119.9 (C-3), 118.6 (C-4c), 115.6 (C-11), 115.4 (C-7b), 114.2 (C-4b), 107.1 (C-1), 91.3 (C-5′), 84.2 (C-4′), 80.3 (C-1′), 60.5 (C-4′OCH_3_), 57.4 (C-3′), 50.5 (C-7), 46.1 (C-3′NCH_3_), 21.2 (C-6′), 14.3 (C-2′); LR-ESI-MS *m*/*z* 467.3 [M+H]^+^.

### 3.4. Cell Culture Method

Patients with NPs were recruited, and NPDFs were cultivated as previously reported [49]. The study was approved by the local ethics committee of Inje University, Busan Paik Hospital, Busan, Republic of Korea (Approval code: 80/2020). The purity of the NPDFs was checked against a panel of antibodies of fibroblast marker (cat. No. ab254015, Abcam Inc., Cambridge, MA, USA) and characteristic cell morphology data. NPDFs were used for 4th to 6th cell passages. 

### 3.5. Cytotoxicity

The cell viability was assessed using the CCK-8 assay. NPDFs (1 × 10^5^ cells/well) were cultured in 96-well microplates using Dulbecco’s Modified Eagle Medium (DMEM). The NPDFs were treated with different concentrations of STA (1, 5, and 10 ng/mL). After 24 h of 37 °C (5% humidified CO_2_) incubation, 450 nm absorbance analysis was performed using a microplate reader (SpectraMax M2e, Molecular Devices, Sunnyvale, CA, USA). All tests were carried out in triplicate.

### 3.6. Western Blot Analysis

NPDF lysates were collected in lysis buffer (G-Biosciences, St. Louis, MO, USA) with a protease inhibitor cocktail (Roche Diagnostics, Mannheim, Germany). The same amount of proteins was separated using 10% sodium dodecyl sulfate-polyacrylamide mini-gel electrophoresis and transferred to the nitrocellulose membrane (GE Healthcare Life Sciences, Chalfont, UK). After the night-time incubation of a specific primary antibody (α-SMA, Col-1, fibronectin, and p-Smad 2), the membrane was incubated with a secondary antibody (IgG for goat anti-mouse) conjugated to horseradish peroxidase. Immunoreactive bands were visualized using an enhanced chemiluminescence detection system (Pierce Biotechnology, Inc., Rockford, IL, USA). Band images were captured and analyzed using image systems (AI 600, GE Healthcare Life Sciences, Canton, MA, USA) and ImageJ software (ver. 1.52a; National Institutes of Health, Bethesda, MD, USA).

### 3.7. Ex Vivo Experiments

NP tissues were cultured with the air–liquid interface organ culture method. As Park et al. reported earlier, NP patients were recruited [50]. The study was approved by the Local Ethics Committee of the University of Inje, the Busan Hospital, Busan, Korea. NPs were obtained from the middle of the meatus and cut into small pieces (3 mm^3^). In order to investigate the inhibition of STA on VEGF expression, tissue fragments were saturated in DMEM for 1 h in the presence or absence of STA (10, 30, and 50 μg/mL). The tissue fragments were then placed on hydrated 1 × 1 cm gelatin sponge (Spongostan, Johnson and Johnson, Austin, TX, USA), with the mucosa facing upwards and the submucosa facing downwards. The gelatin sponge on which the NP tissue was placed was inserted into the well of the six wells containing 3 mL of the culture solution. The plates were placed in a humidified CO_2_ incubator of 5% for 24 h.

### 3.8. Statistical Analysis

All the data in the experiments are presented as mean ± standard error of the mean. All statistical analyses were conducted with GraphPad Prism software (version 5.0; GraphPad Software Inc., La Jolla, CA, USA). Dunnett’s multiple range test was used for groups comparisons. *p* < 0.05 was considered statistically significant.

## 4. Conclusions

Although STA is known for its various physiological activities, this study is the first to demonstrate its inhibitory efficacy against the NPs. Through this study, we were able to establish optimal culture conditions to enhance the production of STA by the SNC087 strain and confirm its potential inhibition effect on the NPs. We intend to further investigate the therapeutic potential of STA for the treatment of the NPs through more in-depth research.

We demonstrated that STA effectively suppresses ECM production and myofibroblast differentiation using TGF-β1-induced NPDFs. These inhibitory activities of STA are achieved by regulating the Smad 2 signaling pathway. In addition, STA reduced the VEGF expression level in NP tissues in an organ culture. Through this study, we established optimal culture conditions to enhance the production of by the SNC087 strain and confirm its potential inhibition effect on the generation of NPs. Through more in-depth research, we intend to further investigate the therapeutic potential of STA to inhibit the remodeling of NPs after surgery to remove NPs.

## Data Availability

The data presented in this study are available on request.

## Supplementary Materials

The following supporting information can be downloaded at: https://www.mdpi.com/article/10.3390/md22010039/s1, Figure S1: ^1^H NMR spectrum (400 MHz, CDCl_3_) of Staurosporine (**1**); Figure S2: ^13^C NMR spectrum (100 MHz, CDCl_3_) of Staurosporine (**1**).
