# Supplementary material for: The Inhibition Effect and Mechanism of Staurosporine Isolated from Streptomyces sp. SNC087 Strain on Nasal Polyp
Source: Mar Drugs. 2024 Jan 11;22(1):39. doi: 10.3390/md22010039 (PMC10820969; doi:10.3390/md22010039)

*Supporting Information*

# The Inhibition Effect and Mechanism of Staurosporine Isolated from *Streptomyces* sp. SNC087 Strain on Nasal Polyp

**Grace Choi [1,*], Eun-Young Lee [2], Dawoon Chung [1], Kichul Choi [1], Woon-Jong Yu [1], Sang-Jip Nam [2], Seong-Kook Park [3] and Il-Whan Choi [4,*]**

[1] Department of Microbial Resources, National Marine Biodiversity Institute of Korea, Seocheon, 33662, Republic of Korea
[2] Department of Chemistry and Nanoscience, Ewha Womans University, Seoul, 03760, Republic of Korea
[3] Department of Otorhinolaryngology-Head & Neck Surgery, Busan Paik Hospital, Inje University College of Medicine, Busan 47392, Republic of Korea
[4] Department of Microbiology and Immunology, Inje University College of Medicine, Busan 47392, Republic of Korea
* Correspondence: gchoi@mabik.re.kr, cihima@inje.ac.kr

# Table of Contents

Figure S1 $^{1}H$ NMR spectrum (400 MHz, $CDCl_3$) of Staurosporine (**1**) ········ **S3**

Figure S2 $^{13}C$ NMR spectrum (100 MHz, $CDCl_3$) of Staurosporine (**1**) ········ **S4**

**Figure S1** $^{1}$H NMR spectrum (400 MHz, $CDCl_3$) of Staurosporine (**1**)

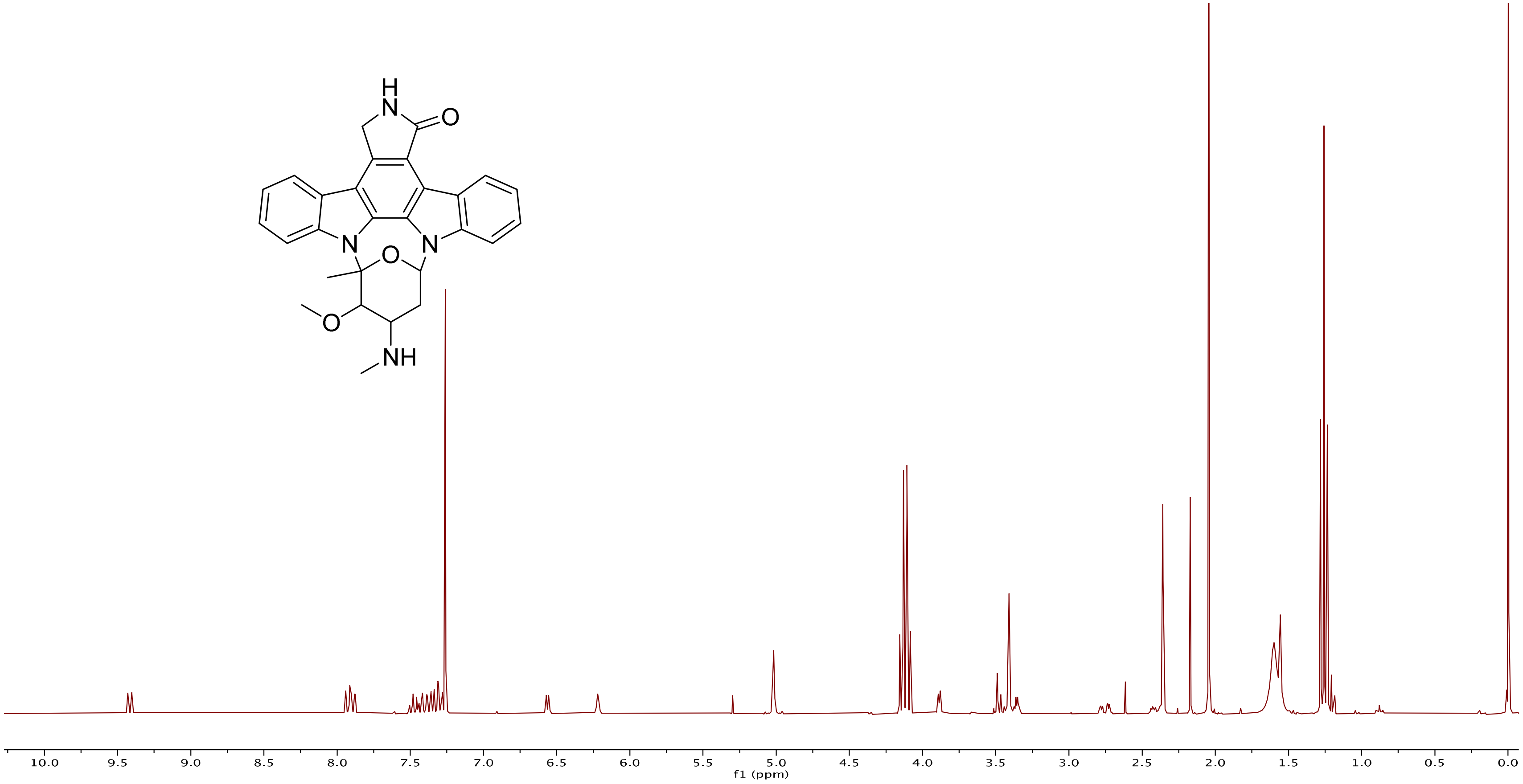

**Figure S2** $^{13}$C NMR spectrum (100 MHz, $CDCl_3$) of Staurosporine (**1**)

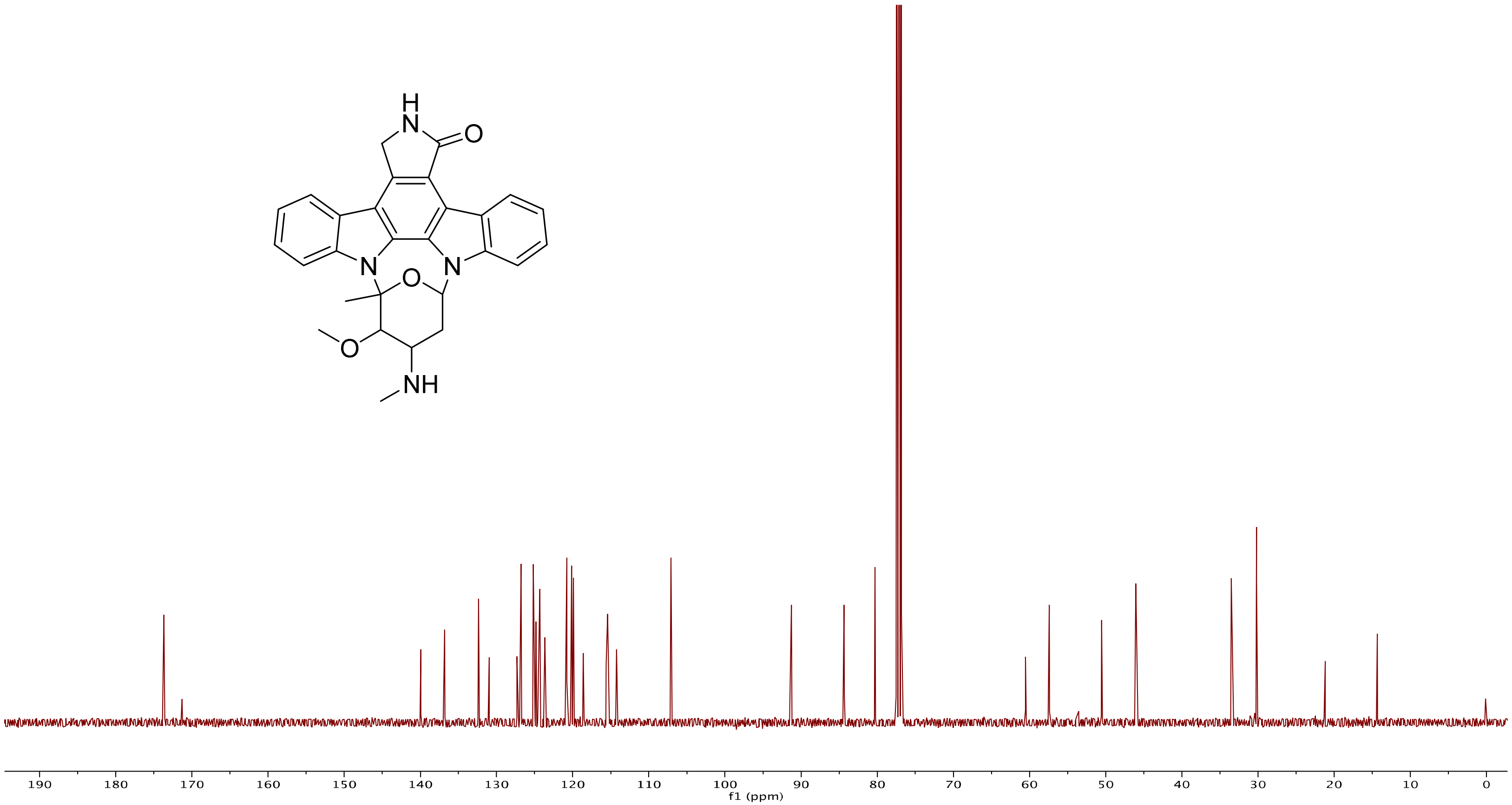

Supplement: Supplementary file 1 [file marinedrugs-22-00039-s001.zip › marinedrugs-2789048-supplementary.pdf]
